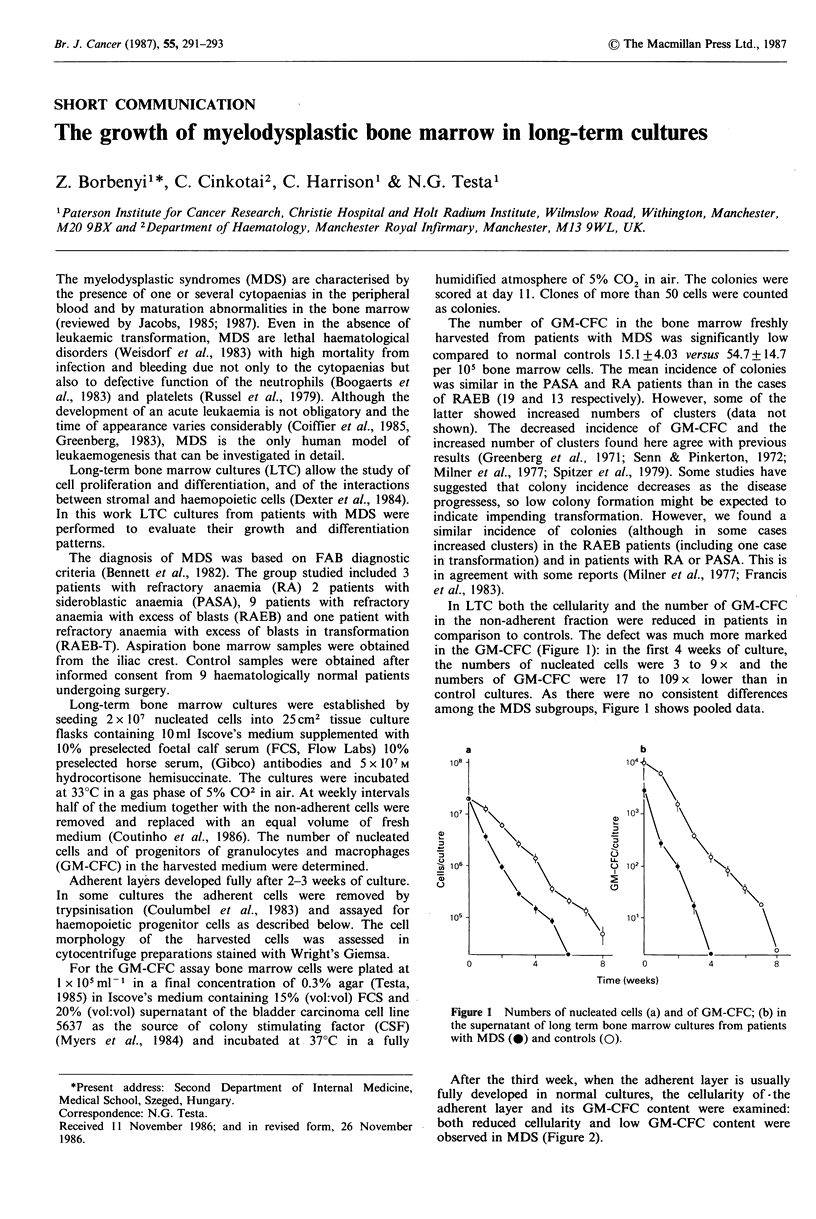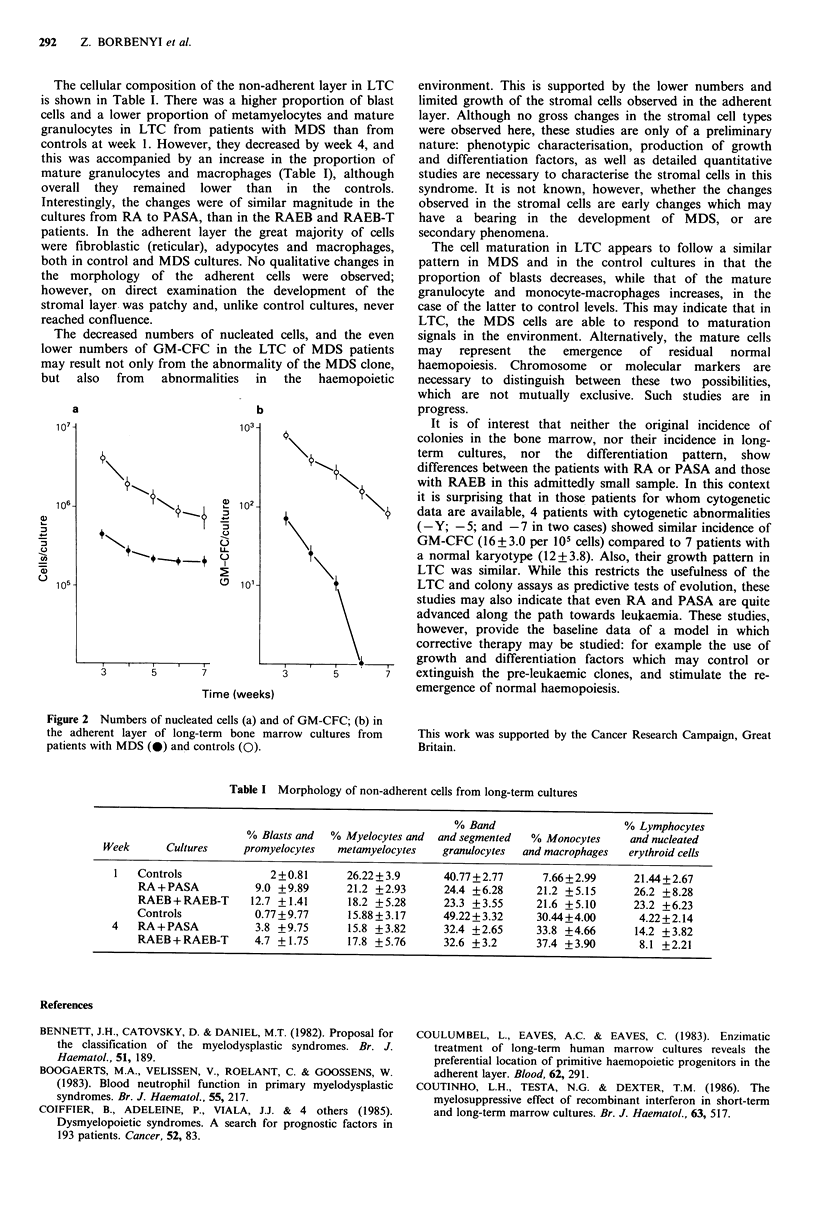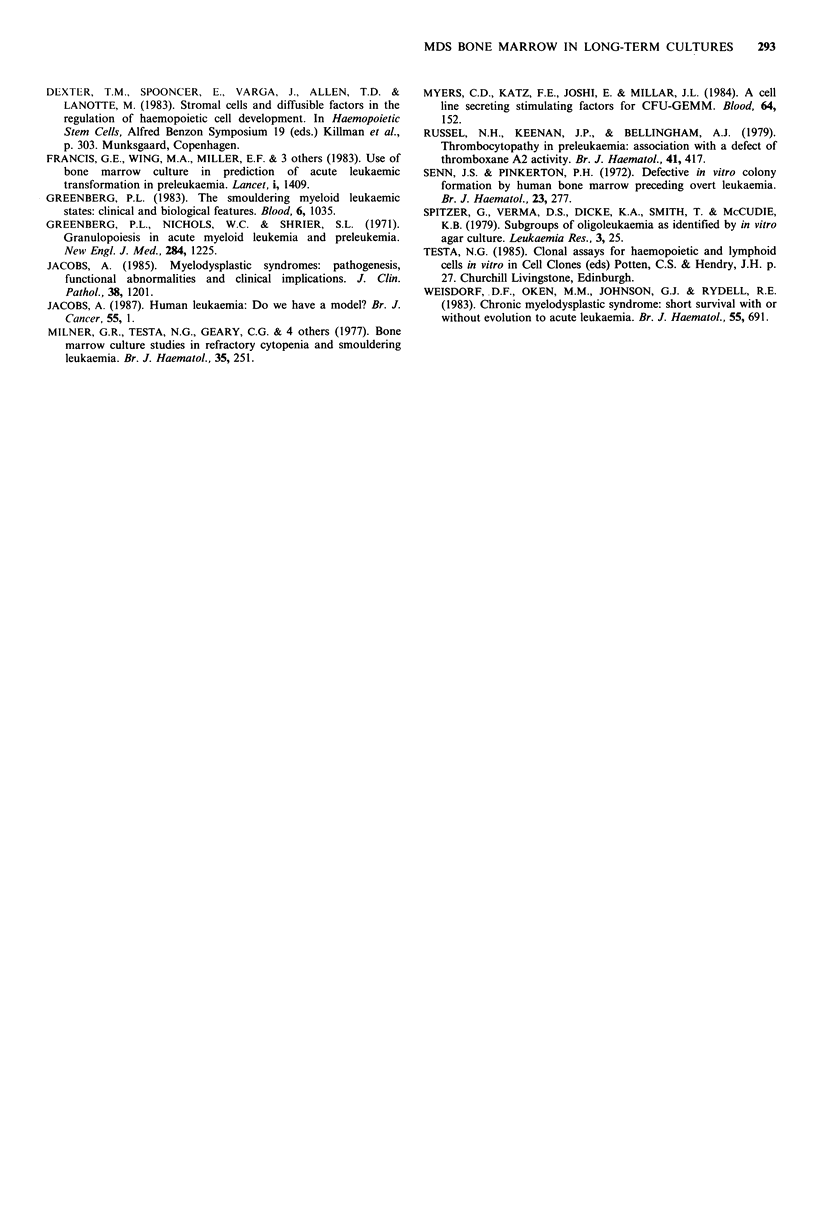# The growth of myelodysplastic bone marrow in long-term cultures.

**DOI:** 10.1038/bjc.1987.56

**Published:** 1987-03

**Authors:** Z. Borbenyi, C. Cinkotai, C. Harrison, N. G. Testa


*Br. J. Cancer* (1987), **55**, 291–293 © The Macmillan Press Ltd., 1987

### SHORT COMMUNICATION

### The growth of myelodysplastic bone marrow in long-term cultures

Z. Borbenyi[1]*, C. Cinkotai[2], C. Harrison[1] & N.G. Testa[1]

[1]*Paterson Institute for Cancer Research, Christie Hospital and Holt Radium Institute, Wilmslow Road, Withington, Manchester, M20 9BX and* [2]*Department of Haematology, Manchester Royal Infirmary, Manchester, M13 9WL, UK.*

The myelodysplastic syndromes (MDS) are characterised by the presence of one or several cytopaenias in the peripheral blood and by maturation abnormalities in the bone marrow (reviewed by Jacobs, 1985; 1987). Even in the absence of leukaemic transformation, MDS are lethal haematological disorders (Weisdorf *et al.*, 1983) with high mortality from infection and bleeding due not only to the cytopaenias but also to defective function of the neutrophils (Boogaerts *et al.*, 1983) and platelets (Russel *et al.*, 1979). Although the development of an acute leukaemia is not obligatory and the time of appearance varies considerably (Coiffier *et al.*, 1985, Greenberg, 1983), MDS is the only human model of leukaemogenesis that can be investigated in detail.

Long-term bone marrow cultures (LTC) allow the study of cell proliferation and differentiation, and of the interactions between stromal and haemopoietic cells (Dexter *et al.*, 1984). In this work LTC cultures from patients with MDS were performed to evaluate their growth and differentiation patterns.

The diagnosis of MDS was based on FAB diagnostic criteria (Bennett *et al.*, 1982). The group studied included 3 patients with refractory anaemia (RA) 2 patients with sideroblastic anaemia (PASA), 9 patients with refractory anaemia with excess of blasts (RAEB) and one patient with refractory anaemia with excess of blasts in transformation (RAEB-T). Aspiration bone marrow samples were obtained from the iliac crest. Control samples were obtained after informed consent from 9 haematologically normal patients undergoing surgery.

Long-term bone marrow cultures were established by seeding $2 \times 10^7$ nucleated cells into $25\,cm^2$ tissue culture flasks containing 10 ml Iscove's medium supplemented with 10% preselected foetal calf serum (FCS, Flow Labs) 10% preselected horse serum, (Gibco) antibodies and $5 \times 10^7$ M hydrocortisone hemisuccinate. The cultures were incubated at 33°C in a gas phase of 5% $CO^2$ in air. At weekly intervals half of the medium together with the non-adherent cells were removed and replaced with an equal volume of fresh medium (Coutinho *et al.*, 1986). The number of nucleated cells and of progenitors of granulocytes and macrophages (GM-CFC) in the harvested medium were determined.

Adherent layers developed fully after 2–3 weeks of culture. In some cultures the adherent cells were removed by trypsinisation (Coulumbel *et al.*, 1983) and assayed for haemopoietic progenitor cells as described below. The cell morphology of the harvested cells was assessed in cytocentrifuge preparations stained with Wright's Giemsa.

For the GM-CFC assay bone marrow cells were plated at $1 \times 10^5\,ml^{-1}$ in a final concentration of 0.3% agar (Testa, 1985) in Iscove's medium containing 15% (vol:vol) FCS and 20% (vol:vol) supernatant of the bladder carcinoma cell line 5637 as the source of colony stimulating factor (CSF) (Myers *et al.*, 1984) and incubated at 37°C in a fully humidified atmosphere of 5% $CO_2$ in air. The colonies were scored at day 11. Clones of more than 50 cells were counted as colonies.

The number of GM-CFC in the bone marrow freshly harvested from patients with MDS was significantly low compared to normal controls $15.1 \pm 4.03$ *versus* $54.7 \pm 14.7$ per $10^5$ bone marrow cells. The mean incidence of colonies was similar in the PASA and RA patients than in the cases of RAEB (19 and 13 respectively). However, some of the latter showed increased numbers of clusters (data not shown). The decreased incidence of GM-CFC and the increased number of clusters found here agree with previous results (Greenberg *et al.*, 1971; Senn & Pinkerton, 1972; Milner *et al.*, 1977; Spitzer *et al.*, 1979). Some studies have suggested that colony incidence decreases as the disease progressess, so low colony formation might be expected to indicate impending transformation. However, we found a similar incidence of colonies (although in some cases increased clusters) in the RAEB patients (including one case in transformation) and in patients with RA or PASA. This is in agreement with some reports (Milner *et al.*, 1977; Francis *et al.*, 1983).

In LTC both the cellularity and the number of GM-CFC in the non-adherent fraction were reduced in patients in comparison to controls. The defect was much more marked in the GM-CFC (Figure 1): in the first 4 weeks of culture, the numbers of nucleated cells were 3 to 9× and the numbers of GM-CFC were 17 to 109× lower than in control cultures. As there were no consistent differences among the MDS subgroups, Figure 1 shows pooled data.

**Figure 1** Numbers of nucleated cells (a) and of GM-CFC; (b) in the supernatant of long term bone marrow cultures from patients with MDS (●) and controls (○).

After the third week, when the adherent layer is usually fully developed in normal cultures, the cellularity of the adherent layer and its GM-CFC content were examined: both reduced cellularity and low GM-CFC content were observed in MDS (Figure 2).

*Present address: Second Department of Internal Medicine, Medical School, Szeged, Hungary.

Correspondence: N.G. Testa.

Received 11 November 1986; and in revised form, 26 November 1986.

The cellular composition of the non-adherent layer in LTC is shown in Table I. There was a higher proportion of blast cells and a lower proportion of metamyelocytes and mature granulocytes in LTC from patients with MDS than from controls at week 1. However, they decreased by week 4, and this was accompanied by an increase in the proportion of mature granulocytes and macrophages (Table I), although overall they remained lower than in the controls. Interestingly, the changes were of similar magnitude in the cultures from RA to PASA, than in the RAEB and RAEB-T patients. In the adherent layer the great majority of cells were fibroblastic (reticular), adypocytes and macrophages, both in control and MDS cultures. No qualitative changes in the morphology of the adherent cells were observed; however, on direct examination the development of the stromal layer was patchy and, unlike control cultures, never reached confluence.

The decreased numbers of nucleated cells, and the even lower numbers of GM-CFC in the LTC of MDS patients may result not only from the abnormality of the MDS clone, but also from abnormalities in the haemopoietic

**Figure 2** Numbers of nucleated cells (a) and of GM-CFC; (b) in the adherent layer of long-term bone marrow cultures from patients with MDS (●) and controls (○).

environment. This is supported by the lower numbers and limited growth of the stromal cells observed in the adherent layer. Although no gross changes in the stromal cell types were observed here, these studies are only of a preliminary nature: phenotypic characterisation, production of growth and differentiation factors, as well as detailed quantitative studies are necessary to characterise the stromal cells in this syndrome. It is not known, however, whether the changes observed in the stromal cells are early changes which may have a bearing in the development of MDS, or are secondary phenomena.

The cell maturation in LTC appears to follow a similar pattern in MDS and in the control cultures in that the proportion of blasts decreases, while that of the mature granulocyte and monocyte-macrophages increases, in the case of the latter to control levels. This may indicate that in LTC, the MDS cells are able to respond to maturation signals in the environment. Alternatively, the mature cells may represent the emergence of residual normal haemopoiesis. Chromosome or molecular markers are necessary to distinguish between these two possibilities, which are not mutually exclusive. Such studies are in progress.

It is of interest that neither the original incidence of colonies in the bone marrow, nor their incidence in long-term cultures, nor the differentiation pattern, show differences between the patients with RA or PASA and those with RAEB in this admittedly small sample. In this context it is surprising that in those patients for whom cytogenetic data are available, 4 patients with cytogenetic abnormalities (−Y; −5; and −7 in two cases) showed similar incidence of GM-CFC (16±3.0 per $10^5$ cells) compared to 7 patients with a normal karyotype (12±3.8). Also, their growth pattern in LTC was similar. While this restricts the usefulness of the LTC and colony assays as predictive tests of evolution, these studies may also indicate that even RA and PASA are quite advanced along the path towards leukaemia. These studies, however, provide the baseline data of a model in which corrective therapy may be studied: for example the use of growth and differentiation factors which may control or extinguish the pre-leukaemic clones, and stimulate the re-emergence of normal haemopoiesis.

This work was supported by the Cancer Research Campaign, Great Britain.

**Table I** Morphology of non-adherent cells from long-term cultures

| *Week* | *Cultures* | *% Blasts and promyelocytes* | *% Myelocytes and metamyelocytes* | *% Band and segmented granulocytes* | *% Monocytes and macrophages* | *% Lymphocytes and nucleated erythroid cells* |
|---|---|---|---|---|---|---|
| 1 | Controls | 2±0.81 | 26.22±3.9 | 40.77±2.77 | 7.66±2.99 | 21.44±2.67 |
| | RA+PASA | 9.0 ±9.89 | 21.2 ±2.93 | 24.4 ±6.28 | 21.2 ±5.15 | 26.2 ±8.28 |
| | RAEB+RAEB-T | 12.7 ±1.41 | 18.2 ±5.28 | 23.3 ±3.55 | 21.6 ±5.10 | 23.2 ±6.23 |
| | Controls | 0.77±9.77 | 15.88±3.17 | 49.22±3.32 | 30.44±4.00 | 4.22±2.14 |
| 4 | RA+PASA | 3.8 ±9.75 | 15.8 ±3.82 | 32.4 ±2.65 | 33.8 ±4.66 | 14.2 ±3.82 |
| | RAEB+RAEB-T | 4.7 ±1.75 | 17.8 ±5.76 | 32.6 ±3.2 | 37.4 ±3.90 | 8.1 ±2.21 |

### References

BENNETT, J.H., CATOVSKY, D. & DANIEL, M.T. (1982). Proposal for the classification of the myelodysplastic syndromes. *Br. J. Haematol.*, **51**, 189.

BOOGAERTS, M.A., VELISSEN, V., ROELANT, C. & GOOSSENS, W. (1983). Blood neutrophil function in primary myelodysplastic syndromes. *Br. J. Haematol.*, **55**, 217.

COIFFIER, B., ADELEINE, P., VIALA, J.J. & 4 others (1985). Dysmyelopoietic syndromes. A search for prognostic factors in 193 patients. *Cancer*, **52**, 83.

COULUMBEL, L., EAVES, A.C. & EAVES, C. (1983). Enzimatic treatment of long-term human marrow cultures reveals the preferential location of primitive haemopoietic progenitors in the adherent layer. *Blood*, **62**, 291.

COUTINHO, L.H., TESTA, N.G. & DEXTER, T.M. (1986). The myelosuppressive effect of recombinant interferon in short-term and long-term marrow cultures. *Br. J. Haematol.*, **63**, 517.

DEXTER, T.M., SPOONCER, E., VARGA, J., ALLEN, T.D. & LANOTTE, M. (1983). Stromal cells and diffusible factors in the regulation of haemopoietic cell development. In *Haemopoietic Stem Cells,* Alfred Benzon Symposium 19 (eds.) Killman *et al.,* p. 303. Munksgaard, Copenhagen.

FRANCIS, G.E., WING, M.A., MILLER, E.F. & 3 others (1983). Use of bone marrow culture in prediction of acute leukaemic transformation in preleukaemia. *Lancet,* **i,** 1409.

GREENBERG, P.L. (1983). The smouldering myeloid leukaemic states: clinical and biological features. *Blood,* **6,** 1035.

GREENBERG, P.L., NICHOLS, W.C. & SHRIER, S.L. (1971). Granulopoiesis in acute myeloid leukemia and preleukemia. *New Engl. J. Med.,* **284,** 1225.

JACOBS, A. (1985). Myelodysplastic syndromes: pathogenesis, functional abnormalities and clinical implications. *J. Clin. Pathol.,* **38,** 1201.

JACOBS, A. (1987). Human leukaemia: Do we have a model? *Br. J. Cancer,* **55,** 1.

MILNER, G.R., TESTA, N.G., GEARY, C.G. & 4 others (1977). Bone marrow culture studies in refractory cytopenia and smouldering leukaemia. *Br. J. Haematol.,* **35,** 251.

MYERS, C.D., KATZ, F.E., JOSHI, E. & MILLAR, J.L. (1984). A cell line secreting stimulating factors for CFU-GEMM. *Blood,* **64,** 152.

RUSSEL, N.H., KEENAN, J.P., & BELLINGHAM, A.J. (1979). Thrombocytopathy in preleukaemia: association with a defect of thromboxane A2 activity. *Br. J. Haematol.,* **41,** 417.

SENN, J.S. & PINKERTON, P.H. (1972). Defective *in vitro* colony formation by human bone marrow preceding overt leukaemia. *Br. J. Haematol.,* **23,** 277.

SPITZER, G., VERMA, D.S., DICKE, K.A., SMITH, T. & McCUDIE, K.B. (1979). Subgroups of oligoleukaemia as identified by *in vitro* agar culture. *Leukaemia Res.,* **3,** 25.

TESTA, N.G. (1985). Clonal assays for haemopoietic and lymphoid cells *in vitro* in Cell Clones (eds) Potten, C.S. & Hendry, J.H. p. 27. Churchill Livingstone, Edinburgh.

WEISDORF, D.F., OKEN, M.M., JOHNSON, G.J. & RYDELL, R.E. (1983). Chronic myelodysplastic syndrome: short survival with or without evolution to acute leukaemia. *Br. J. Haematol.,* **55,** 691.